# Protective effects of paraoxonase-1, vitamin E and selenium, and oxidative stress index on the susceptibility of low density lipoprotein to oxidation in diabetic patients with/without coronary artery disease

**DOI:** 10.1186/s40001-023-01254-9

**Published:** 2023-08-27

**Authors:** Fatemeh Mehvari, Fatemeh Imanparast, Pegah Mohaghegh, Abbas Alimoradian, Nafiseh Khansari, Behnoosh Ansari Asl, Ali Khosrowbeygi

**Affiliations:** 1 Student Research Committee, Arak University of Medical Sciences, Arak, Iran; 2 Department of Biochemistry and Genetics, Faculty of Medicine, Arak University of Medical Sciences, Arak, Iran; 3 Depertment of community medicine school of medicine, Faculty of Medicine, Arak University of Medical Sciences, Arak, Iran; 4 Department of Pharmacology, Faculty of Medicine, Arak University of Medical Sciences, Arak, Iran; 5 A Food and Drug Deputy, Arak University of Medical Sciences, Arak, Iran

**Keywords:** Diabetes mellitus, Coronary artery disease, Oxidative stress, Vitamin E, Selenium, Oxidized low density lipoprotein, Paraoxonase-1

## Abstract

**Background:**

The oxidative modification of low density lipoprotein (LDL) is closely associated with an increased risk for coronary artery disease (CAD) in diabetic patients. The purpose of this study is to investigate the relation between serum vitamin E and selenium, paraoxonase-1 (PON1) activity, total antioxidant capacity (TAC), total oxidant status (TOS), malondialdehyde (MDA), and oxidative stress index (OSI) values with the susceptibility of LDL to oxidative modification and the possibility of CAD in diabetic patients.

**Method:**

This study was designed as a case control survey of 82 diabetes patients divided into two groups including T2DM alone (as group I) and both T2DM and CAD (as group II). Fasting blood samples were taken to the assay of fasting blood glucose (FBG), HbA1c, total cholesterol (TC), TAC, TOS, MDA, OSI, vitamin E, selenium, oxidized low density lipoprotein (Ox-LDL), and activity of PON1.

**Results:**

Ox-LDL, MDA, TOS, and OSI values in groups II were significantly higher compared with group I (all with *P* value = 0.000). TAC, vitamin E, selenium, and PON1 activity values were significantly lower in group II compared with groups I (*P* value = 0.000; *P* value = 0.000; *P* value = 0.007; *P* value = 0.003, respectively). There were significant relationships between the amounts of TAC, TOS, OSI, and vitamin E with the amounts of PON1 activity and Ox-LDL (*p* < 0.05). But Ox-LDL and PON1 activity correlated weakly with together (*p* = 0.094).

**Conclusion:**

Results of this study support the belief that oxidative stress might be an important etiologic factor which makes some diabetics more susceptible to CAD. Increased oxidative stress may be a potential therapeutic target in the prevention and management of CAD in diabetic patients.

## Background

Diabetes mellitus (DM) is defined as a disorder characterized by insufficient production of insulin or lack of normal response to it in body, leading blood glucose level to be abnormally high [1]. In type 2 DM (T2DM), the blood glucose level elevates due to resistant main regulating organs including liver, fat, and muscles to insulin and prevent the entry of glucose into cells of these tissues [2].

Diabetes is associated with both microvascular and macrovascular complications including nephropathy, neuropathy, and diabetic retinopathy (microvascular) and ischemic heart disease, coronary artery disease (CAD), peripheral vascular disease, and cerebrovascular disease (macrovascular) [3, 4]. The main pathologic mechanism of CAD resulting from diabetes is atherosclerosis. In the pathogenesis of atherosclerosis disease, oxidative stress is one of the mechanisms of the effects of many genetic, biochemical and nutritional risk factors in endothelium damage, the entry of low density lipoprotein (LDL) into the intima, its oxidation, the expression of receptors on the surface of macrophages, fatty plaques formation, and the proliferation of muscle cells as the most important molecular changes leading this disease [5–7].

Various forms of the modified lipoproteins such as oxidized LDL (Ox-LDL), oxidized high density lipoprotein (Ox-HDL), nitrated LDL, and nitrated HDL play an important role in the development of atherosclerosis [8]. Ox-LDL leads to the progression of lesions of atherosclerosis in diabetes mellitus (DM), aging, obesity, non-alcoholic fatty liver, metabolic syndrome, CVD, cerebrovascular diseases, nephrotic syndrome, chronic renal failure, diabetic nephropathy, nephrosclerosis and acute renal failure. It causes and plays an important role in the pathogenesis of these diseases [9, 10]. Ox-LDL have a wide range of biological properties such as activation and proliferation of monocytes/ macrophages in the arterial wall, the increasing of expression of platelet-derived growth factor and basic fibroblast growth factor, stimulation of adhesion and accumulation of platelets by reducing endothelial nitric oxide production, increasing prostacyclin production, the stimulating of prostaglandins synthesis, and stimulation of collagen production by SMCs by which appear to promote atherosclerosis [11].

Human paraoxonases (PON) are the calcium-dependent lactonases including PON1, PON2, and PON3. In the circulation, PON1 is almost exclusively associated with HDL. Such communication is carried out by the phospholipids of the HDL surface. Protective role of PON-1 on LDL against oxidation is accompanied by inactivation of itself. PON1 has special benefit against atherosclerosis by prevention of Ox-LDL formation and removal of Ox-LDL associated oxidized lipids which are generated during LDL oxidation [12].

In recent years, special attention has been paid to the use of various antioxidants in reducing oxidative stress in various diseases, especially diabetes [13]. Vitamin E as the major fat-soluble antioxidant in LDL particles, increases LDL resistance to oxidation by preventing the lipid peroxidation of polyunsaturated fatty acids and modification of proteins in LDL by ROSs. Also it decreases the uptake of Ox-LDL particles by macrophages by reducing the expression of receptors as CD38 [14]. Selenium is an essential compound for the activity of glutathione peroxidase and selenoprotein, these proteins control blood sugar and reduce oxidative stress and inflammation by increasing insulin sensitivity. Studies also show that selenium and selenoproteins may block many key processes in the development of atherosclerosis, including ROS-induced oxidative stress, ROS-induced LDL oxidation, inflammation (especially monocyte adhesion and migration, cell formation foam, abnormal eicosanoid metabolism), endothelial dysfunction, vascular cell apoptosis, and vascular calcification [15, 16].

In present studies, Ox-LDL in diabetic people is higher than in healthy people, and in diabetic patients with CAD is more than patients without CAD, and Ox-LDL has been introduced as a biomarker for CAD [17]. No study has demonstrated the correlation between serum vitamin E and selenium levels, PON1 activity, total antioxidant capacity (TAC), total oxidant status (TOS), malondialdehyde (MDA), and oxidative stress index (OSI) and the susceptibility of LDL to oxidative modification and the possibility of CAD in diabetic patients. The purpose of this study is to investigate the above-mentioned items.

## Materials and methods

### Subjects

A case control study was conducted in the Imam Reza Specialized Clinic Arak University of Medical Science (Arak, Iran) from August 2021 to February 2023 included 41 patients with definitive T2DM without CAD diagnosis (group I) and 41 patients definitive T2DM with CAD (group II), according to biochemical parameters, history of heart attack, and coronary angiography.

The inclusion criteria involved age between 40 and 60 years, the disease duration for a minimum of 5 years and a maximum of 15 years prior to initiation of the study.

T2DM was identified with an FBS concentration more or equal to 126 mg/dl and HbA1c more or equal to 6.5%, which is mentioned in the American Diabetes Association guidelines. The angiography was used for the classification of T2DM subjects with CAD.

The patients with T2DM received a fixed dose of glibenclamide or repaglinide + metformin and/ or metformin alone for at least 6 months before screening. There should not be significant nutritional changes between the two groups during least 6 months before screening.

The exclusion criteria involved need to use insulin, patients with a history of acute myocardial infarction, percutaneous transluminal coronary angioplasty, infectious or inflammatory disease, severe liver or renal disease, neoplasm, and hematologic disorders, receiving vitamin supplements, essential elements such as zinc and antioxidants during the previous three months, alcohol consumption, smoking, pregnancy and breastfeeding and allergies.

We calculated the sample size according to the following formula for comparison means between two independent groups:

$$N = 2 \times \left( {(Z_{1 - (\alpha /2)} + Z_{1 - \beta } } \right)^{2} \times {{\left( {SD_{1}^{2} + SD_{2}^{2} } \right)} \mathord{\left/ {\vphantom {{\left( {SD_{1}^{2} + SD_{2}^{2} } \right)} {\left( {\overline{X}_{1} - \overline{X}_{2} } \right)}}} \right. \kern-0pt} {\left( {\overline{X}_{1} - \overline{X}_{2} } \right)}}^{2} ,$$

where standard deviation of group I (S1) = 77.5, standard deviation of group II (S2) = 95.9, difference between means (d) = 18.4, type I error of 5% (α) = 0.05, type II error of 20% (β) = 0.2, and z score (z) = 1.96, and serum Ox-LDL (U/L) concentration in diabetic and control participants is a key variable in the study Ganjifrockwala et al. in 2017 [18]. We reached the sample size of 41 persons for each group.

### Ethical and safety considerations

Ethical approval for this study was obtained from the Committee on Human Research, Publication, and Ethics (CHRPE) at Arak University of Medical Sciences, Arak, Iran (IR.ARAKMU.REC.1400.120). All participants completed a written consent for these experiments.

### Biochemical assessments

Blood samples of all subjects were taken after at least a 12-h fasting. Serums were saved after centrifugation (20 min; 3000 rpm) at –80 °C. Serum fasting blood glucose (FBG) and total cholesterol (TC) levels were measured by an enzymatic method (Pars Azmoon Co. kit, Tehran, Iran) using a Liasys autoanalyzer. HbA1c on whole blood was determined by HbA1c point-of-care testing (HbA1c care analyzer, Germany). FBG, TC and HbA1c values were analyzed on the same day of sampling.

The serum values of Ox-LDL (ZellBio, Germany) were measured by enzyme-linked immunosorbent (ELISA) method using detection kit as carried out according to the manufacturer’s instruction.

The levels of serum TAC (Navand, Iran), TOS (Navand, Iran), MDA, (Navand, Iran) and PON1 activity (ZellBio, Germany) were measured by colorimetric tests using detection kits as carried out according to the manufacturer’s instruction.

The serum level of vitamin E (α-tocopherol) was measured by high performance liquid chromatography (HPLC) (KNAUER, Berlin, Germany) method, as previously described[19].

The serum level of selenium was measured by atomic absorption spectroscopy (AAS).

### Statistical analysis

The software package used to analyze the data was SPSS version 17 (SPSS, Chicago, IL, USA). Quantitative variables were described using mean ± standard deviation (SD) and qualitative variables were described using frequency and percentage. Normal distribution of numerical date was assessed by Kolmogorov–Smirnov test. Independent T-test were used for comparison of continuous variables between two groups (T2DM patients with and without CAD) of the study. Correlation between continuous variables (serum Ox-LDL and/or PON1 with TAC, TOS, MDA, vitamin E, and selenium) was assessed using Pearson correlation the study. P value less than 0.05 was considered as significant threshold.

## Results

Table 1 indicates the general characteristics in T2DM patients without CAD (group I) and T2DM patients with CAD (group II). There was no statistically significant difference in the general characteristics including age, BMI, the duration of diabetes, and sex (male/ female) between groups I and II.

**Table 1:** The general variables in T2DM patients with/ without CAD

	T2DM (M ± SD)	T2DM patients with CAD (M ± SD) (n = 41)	*P* value
	(*n* = 41)		
Sex ratio (M/F)	22/19	23/18	
Age (year)	53.66 ± 5.78	55.85 ± 3.78	0.171
BMI (kg/m^2^)	28.62 ± 5.17	27.93 ± 3.74	0.494
Duration of diabetes (year)	8.81 ± 4.15	9.53 ± 3.41	0.149

Data are presented as mean value and standard deviation (SD); indicator the presence or absence of a significant difference between the values of similar variables in the two groups (*P* value). Type 2 diabetes mellitus (T2DM); coronary artery disease (CAD); male/female (M/F); body mass index (BMI).

Regarding the biochemical parameters, significant difference was found in the mean of HbA1c level between groups I and II (7.463 ± 1.219 vs 8.122 ± 1.182; P value = 0.015, respectively), while no significant differences were found in the mean of FBG and TC levels between them (155.70 ± 43.82 vs 145.65 ± 26.92; 158.03 ± 40.41 vs 170.68 ± 33.43; *P* value = 0.214, *P* value = 0.127, respectively) (Table 2).

**Table 2:** The biochemical parameters in T2DM patients with/ without CAD

	T2DM (M ± SD) (*n* = 41)	T2DM patients with CAD(M ± SD) (n = 41)	*P* value
HbA1c (%)	7.46 ± 1.22	8.12 ± 1.18	0.015
FBG (mg/dl)	155.70 ± 43.82	145.64 ± 26.92	0.214
TC(mg/dl)	158.03 ± 40.41	170.68 ± 33.43	0.127
TAC (μmol Trolox equiv./l)	1.18 ± 0.30	0.87 ± 0.24	0.000
TOS (µmol H_2_O_2_equiv/l)	0.64 ± 0.49	1.4 ± 0.9	0.000
OSI (TOS/TAC)	0.60 ± 0.53	1.73 ± 1.22	0.000
MDA(mmol/l)	21 ± 3.18	26.76 ± 6.97	0.000
PON1 activity	48.29 ± 11.47	39.76 ± 14.03	0.003
Ox-LDL(ng/ml)	1276.56 ± 271.31	1498.17 ± 159.82	0.000
Vitamin E (U/ml)	4.61 ± 2.01	2.65 ± 0.84	0.000
Selenium (ppb)	24.08 ± 6.80	18.64 ± 6.38	0.000

Data are presented as mean value and standard deviation (SD); indicator the presence or absence of a significant difference between the values of similar variables in the two groups (P value). Type 2 diabetes mellitus (T2DM); coronary artery disease (CAD); fasting blood glucose (FBG); hemoglobin A1C (HbA1c); total cholesterol (TC); total antioxidant capacity (TAC); total oxidant power (TOS); malondialdehyde (MDA); vitamin E; selenium (Se); oxidized low density lipoprotein (Ox-LDL); paraoxonases-1 (PON1)

The results showed that the value of TAC was significantly lower in group II compared to the group I (0.8783 ± 0.24058 vs 1.1786 ± 0.30191; *P* value = 0.000, respectively), while the results showed that the values of TOS, OSI, and MDA as the oxidants markers were significantly higher in group II compared to group I (1.3983 ± 0.89930 vs 0.6425 ± 0.49794: *p* = 0.000; 1.73 ± 1.22 vs 0.60 ± 0.53: *p* = 0.000; 26.7632 ± 6.96692 vs 20.9990 ± 3.18162: *P* value = 0.000, respectively). There was statistically significant difference in the activity of PON1 between groups I and II with decrease in group II (39.7637 ± 14.03156 vs 48.2961 ± 11.46858: *P* value = 0.003, respectively). Serum Ox-LDL levels were significantly increased in group II than group I (1498.1707 ± 159.81926 vs 1276.5610 ± 271.30776: *P* value = 0.000, respectively) (Table 2).

Table 2 indicates the values of vitamin E and selenium as two antioxidant micronutrients that were significantly lower in group II compared to group I (2.6510 ± 0.84501 vs 4.6093 ± 2.01485; 18.6478 ± 6.38080 vs 24.0817 ± 6.80693: *P* value = 0.000, P value = 0.000, respectively).

There was a significant inverse relationship between PON 1 activity with TOS value in the whole study population (*P* value = 0.05 and *P* value = 0.002, respectively). Also, there was a significant direct relationship between PON 1 activity with TAC and vitamin E in the whole study population (*P* value = 0.016 and *P* value = 0.013, respectively). PON1 activity had a direct relationship with selenium and an inverse relationship with MDA and Ox-LDL in the whole study population, which were not significant (Table 3).

**Table 3:** Relation between the biochemical parameters in the whole study population (*N* = 82)

The whole study population (*N* = 82)				
	Ox-LDL		PON1	
	*R*	*p*	*R*	*P*
TAC	− 0.361	0.001	0.265	0.016
TOS	0.347	0.001	− 0.217	0.05
OSI	0.332	0.002	− 0.231	0.037
MDA	0.187	0.093	− 0.343	0.002
Selenium	− 0.025	0.877	0.142	0.204
Vitamin E	− 0.382	0.000	0.273	0.013
HbA1c	0.203	0.068	− 0.033	0.769
PON1	− 0.186	0.094	–	–
Ox-LDL	–	–	− 0.186	0.094

Pearson’s correlation co-efficient (*r*), sign indicates negative (inverse) correlation (–), sign indicates positive correlation ( +), indicator the presence or absence of a significant difference between serum Ox-LDL and/or PON1 with TAC, TOS, MDA, vitamin E, and selenium in all diabetic patients with/without CAD (P)

There was a significant direct relationship between Ox-LDL value with TOS (*P* value = 0.001). Also, there was a significant inverse relationship between Ox-LDL activity with TAC and vitamin E in the whole study population (*P* value = 0.000 and *P* value = 0.01, respectively). Ox-LDL had a weak direct relationship with MDA (*P* value = 0.093) and a weak inverse relationship with PON1 in the whole study population (*P* value = 0.094) (Table 3). While Ox- LDL itself correlated only weakly with HbA1c (*P* value = 0.068, respectively).

## Discussion

Although CAD in T2DM patients is considered as one of its complications, some of them are more susceptible to this complication. Evidence shows that Ox-LDL has important role in atherogenesis by promoting inflammation and lipid deposition in the arterial wall [17, 20–22].

In this study, we observed that higher Ox-LDL associated with diabetic patients with CAD than these patients without CAD. EL- Bassiouni et al. in 2022 showed Ox-LDL levels were significantly higher in diabetics with CAD than patients without CAD [22]. Also, Xu et al. in 2022 reported that Ox-LDL/LDL-C ratio associated with severity of diabetic coronary lesions and significantly increased in patients with severe coronary lesions [17].

In our study, HbA1c was higher in diabetic patients with CAD than diabetic patients without CAD. While in Xu's study, this increase in amount was not seen, and even in some groups with CAD, such as severe obstruction, the amount is lower than that of diabetics without CAD, which was the reason for insulin injection by this group [17], while in our study, people injecting insulin were excluded from the study. The results of Khan's study in 2021 were completely opposite to Xu's results and the amount of HbA1c in diabetic patients with CAD is more and high HbA1c was related to severe CAD [23].

In study of Njajou et al. in 2009 reported an association between Ox-LDL and HbA1c [24]. In our study (*P* value = 0.068) similar to Harmon's study in 2016 (*P* value = 0.062), there was weak correlation between Ox-LDL and HbA1c [25]. Hussein et al. in 2007 reported the susceptibility of LDL to oxidative modification in diabetic patients contributes to increased levels of blood HbA1c [26]. This contradiction may be due to the HbA1c values, hence in the Hussein's study this relationship was directly related in HbA1c < 7.3, while the HbA1c means of both groups in our study were > 7.4.

PON1 enzyme with its protective effects on lipid peroxidation as LDL decreases CAD risk in T2DM. In this study, the PON1 activity decreased in diabetic patients with CAD than diabetic patients without CAD with same DM duration. Tartan et al. in 2007 reported PON1 activity decreases parallel to increase of DM duration and severity of CAD [27]. Kumar et al. in 2014 reported age-dependent reverse correlation between Ox-LDL and PON1 in rats [28]. But in our study, PON1 correlated only weakly with Ox-LDL (*P* value = 0.094). Perhaps one of the reasons for this weak correlation is that the antioxidant activity of PON1 has been declared to be dependent on the age and duration of diabetes, while in our study there is no significant correlation between PON1 activity and the age or duration of diabetes. Because in this study, patients participated in a limited age range (40–60) and period of diabetes [5–15 years].

In this study, the vitamin E value decreased in diabetic patients with CAD than diabetic patients without CAD. It had significant positive and negative correlation with PON1 and Ox-LDL in the whole study population. Jarvik et al.'s study in 2002 showed that vitamin E and C, with their antioxidant effects, reduce the oxidation effects of cigarette smoking on LDL [29]. Ghaffari et al. in 2011 reported the protective effects of vitamin E on PON1 activity and LDL oxidation in diabetic rats [30]. Although in some studies, vitamin E as both primary and secondary protection against cardiovascular (CV) events has been proposed, but some studies warn against the use of vitamin E supplements and state that high levels of vitamin E may increase the risk of CAD/MI disease [31]. Studies show that the use of vitamin E supplements is not useful for all people and it should be prescribed to some people such as diabetic patients with low levels of vitamin E in their serum to have effective and useful effects [32].

Selenium as an essential trace element into selenoproteins has the antioxidant and anti-inflammatory properties [33]. In our study, it was shown that in T2DM who have CAD, the serum level of selenium is lower than in diabetics without CAD. The associations were independent of traditional risk factors, including dietary and lifestyle factors, BMI, diabetes duration, age, and FBG. In general populations about the relation of selenium concentrations with the risk of T2D and CVD, some studies reported an inverse association [34], whereas others reported a null association or positive association. Our study is consistent with the prospective study of Qiu et al. in 2022 and Long et al. in 2019 that reported a negative relation between the selenium value and increased risk of CAD in diabetic patients [35, 36], while Sotiropoulos et al. in 2011 reported serum selenium levels did not differ between diabetic subjects with and without CAD [37]. Bleys et al. in 2007 showed high serum selenium levels were positively associated with the prevalence of diabetes [38]. There appears to be a U-shaped relationship between blood selenium levels and the risk of diabetes and stroke [39].

The reactive oxygen species (ROS) plays a pivotal role in the pathogenesis of diabetes and its complications such as CAD with activation or inactivation of various signaling pathways involved in the exacerbation (as NF-*κ*B-mediated proinflammatory signals) or prevention (as Nrf2 and Sirt1-mediated antioxidant signals) of diabetic disorders, respectively [40]. Varadhan et al. in 2022 reported the increase of MDA in CAD patients [41]. Bastani et al. in 2018 showed the TAC and MDA values decreased and increased in CAD patients, respectively [42]. In this study, TOS, OSI, and MDA values increased in diabetic patients with CAD than diabetic patients without CAD. Also, TAC value decreased in diabetic patients with CAD than diabetic patients without CAD. There were significant positive and negative correlations between OSI with Ox-LDL and PON1 in the whole study population, respectively.

The limitations of this study include the absence of a control group including healthy individuals, Failure to measure some relevant biochemical factors such as LDL, Ox-HDL and HDL.

We suggest studies with the presence of healthy people and comparing the changes of their oxidative stress factors with diabetics with/ without CAD, clinical intervention studies with vitamin E and selenium supplements and their effects on biochemical factors, and molecular studies and gene polymorphisms of important molecules in the biochemical pathways of CAD as PON1 in diabetic patients.

## Conclusion and future directions

This the first study demonstrating the relationship of MDA, OSI (TAC and TOS), and PON1 in susceptibility of LDL to the oxidation in DM with/without CAD.

According to our results, this study demonstrated increased oxidative stress might be an important etiologic factor which makes some diabetics more susceptible to CAD. Because of that our next study is planned to give our patients various antioxidants as adjuvant therapy and examine their effects on oxidative stress.

According to the available measured data, DM patients are closely associated with changes in the redox status and that is necessary to minimize the oxidative stress in prevention of the various complications of DM. Therefore, further studies in DM patients are required for indications of antioxidant therapy.

## Data Availability

All data used in the current study are available from the corresponding author on reasonable request.

## Abbreviations

LDL: Low density lipoprotein
CAD: Coronary artery disease
PON1: Paraoxonase-1
TAC: Total antioxidant capacity
TOS: Total oxidant power
MDA: Malondialdehyde
OSI: Oxidative stress index
FBG: Fasting blood glucose
TC: Total cholesterol
Ox-LDL: Oxidized low density lipoprotein
