# Correction: Protective effects of paraoxonase-1, vitamin E and selenium, and oxidative stress index on the susceptibility of low density lipoprotein to oxidation in diabetic patients with/without coronary artery disease

**DOI:** 10.1186/s40001-023-01461-4

**Published:** 2023-10-25

**Authors:** Fatemeh Mehvari, Fatemeh Imanparast, Pegah Mohaghegh, Abbas Alimoradian, Nafiseh Khansari, Behnoosh Ansari Asl, Ali Khosrowbeygi

**Affiliations:** 1 Student Research Committee, Arak University of Medical Sciences, Arak, Iran; 2 Department of Biochemistry and Genetics, Faculty of Medicine, Arak University of Medical Sciences, Arak, Iran; 3 Depertment of Community Medicine School of Medicine, Faculty of Medicine, Arak University of Medical Sciences, Arak, Iran; 4 Department of Pharmacology, Faculty of Medicine, Arak University of Medical Sciences, Arak, Iran; 5 Food and Drug Deputy, Arak University of Medical Sciences, Arak, Iran

**Correction: European Journal of Medical Research (2023) 28:300** 10.1186/s40001-023-01254-9

In the Funding section of this article the grant number relating to Arak University of Medical Sciences given for Ali Khosrowbeygi was incorrectly given as 120 and should have been 6327. The original article [1] has been corrected.
